# PROTOCOL: The effectiveness of skills training to increase employment among those experiencing and at risk of homelessness: A systematic review

**DOI:** 10.1002/cl2.1372

**Published:** 2023-12-09

**Authors:** Zijun Li, Mina Ma, Yongqi Yang, Yanfei Li, Ke Guo, Minyan Yang, Guanghua Liu, Kehu Yang

**Affiliations:** ^1^ School of Public Health, Evidence Based Social Science Research Center/Health Technology Assessment Center Lanzhou University Lanzhou China; ^2^ Key Laboratory of Evidence Based Medicine and Knowledge Translation of Gansu Province Lanzhou University Lanzhou China; ^3^ School of Basic Medical Sciences, Evidence Based Medicine Center Lanzhou University Lanzhou China; ^4^ The First Clinical Medical College Lanzhou University Lanzhou China; ^5^ Law School Lanzhou University Lanzhou China

## Abstract

This is the protocol for a Campbell systematic review. The objectives are as follows. We will solve the following questions: (1) What are the characteristics of skills training services for individuals experiencing or at risk of homelessness? (2) How effectively do the different skills training programs improve employment status, work and life skills, or housing stability? (3) What factors are associated with the variation in the effectiveness of skills training services?

## 1 BACKGROUND

### 1.1 The problem, condition or issue

The United Nations Declaration of Human Rights states that all individuals possess the right to housing (United Nations, 2023). However, almost 2 million people have become homeless in high‐income countries over the last decade (Labour and Social Affairs, 2021). The issue of homelessness has been increasing in high‐income countries since 2017 (Labour and Social Affairs, 2021).

As a vulnerable population, homelessness is associated with a higher incidence of sexually transmitted infections (Williams, 2018), chronic disease (Bernstein, 2015), infectious disease (Beijer, 2012), substance abuse (Aldridge, 2018), and psychiatric disorders (Gutwinski, 2021). Their mortality rates are higher than those housed (Frankish, 2005). The elimination of homelessness must be given top priority globally.

Homelessness Handbook lists one cause of homelessness as “a diminished ability to support themselves either through entitlements or conventional or makeshift labor” (Guha, 2008). Individuals experiencing homelessness possess restricted choices for full‐time or part‐time employment, which impacts their physical health, mental health, and capacity to interact socially (Glass, 2006). Lack of occupational training and life skills are the impediments to employment. It reported that homeless people can thrive in the workplace with “opportunity, training, and sustained support” (Stephen, 2013). Researchers from the Department of Labor's seven‐year job training for the homeless discovered a significant improvement in the employment rate, which enhanced housing stability (National Alliance to End Homelessness, 2016).

### 1.2 The intervention

Skills training refers to a structured and organized process of learning and developing specific abilities, competencies, or techniques. For homeless individuals, training in life skills is critically important for their rapid integration into society. Simultaneously, providing necessary vocational training helps homeless individuals improve their employment prospects and gain meaningful employment opportunities for survival. Vocational training typically involves two parts: hard skills building and soft skills development (Homeless Hub, 2021). Hard skills training seeks to build the abilities needed to acquire a job. Hard skills training is usually combined with technical skills and work experience that assists in gaining employment (GCFGlobal, 2023), such as car repair, carpentry, and restaurant work. Soft skills are a set of non‐technical abilities that enable individuals to interact effectively and harmoniously with others (eSoft Skill, 2023). Soft skills training aims to foster individual skills that can be used in any job, such as communication, teamwork, emotional intelligence, problem‐solving, and adaptability (GCFGlobal, 2023). Life skills are indispensable for employment which refers to a set of practical abilities and knowledge that individuals acquire and use to manage their everyday lives. Life skills are made up of three core competencies: (1) Core or fundamental skills, such as ability in reading, writing and calculation. (2) Self‐sufficient living skills like personal hygiene, home cleaning, and financial management. (3) Social skills, such as empathy, collaboration, and conflict resolution (Homeless Hub, 2023).

### 1.3 How the intervention might work

Summarized the way all kinds of programs enhanced the employment rate of individuals suffering or at risk of homelessness in the following:

1. Work‐related hard skills training: It will provide skills specific to a trade, along with certification, which might help the homeless person overcome the employment barrier.
2. Work‐related soft skills training: It will offer a series of training sessions designed to assist individuals experiencing or at risk of homelessness in their job‐seeking efforts. The training encompasses various aspects, including effective communication with colleagues, work‐related problem‐solving, and more. Employers often prioritize candidates who exhibit qualities such as morality, resourcefulness, and self‐direction. In addition to soft skills development, this training also provides job search guidance, assistance with resume preparation, and interview coaching. These resources aim to enhance the employability of individuals facing or at risk of homelessness, increasing their chances of securing employment.
3. Life skills training: It will cultivate a variety of skills and knowledge that are essential for living independently, like personal hygiene, time management, financial literacy, and so on. The lack of these skills is common among people who are homeless, either because they never acquired them or lost them. Providing life skills to those experiencing homelessness will enable them to reintegrate into society and end their homelessness.

### 1.4 Why it is important to do this review

In the US, since the New Deal's main labor initiatives, there has been a long history of viewing skills training as a solution to the unemployment issue (Wallis, 1988). The Workforce Innovation and Opportunity Act (WIOA) declared that Individuals Training Accounts will support skills training to help youngsters and others with employment dilemmas (US Department of Labor, 2023). The Veterans' Employment & Training Service under the Department of Labor is in charge of implementing multiple initiatives to end veteran homelessness through various programs (Veterans' Employment and Training Service, 2023). There are also some programs available for people facing homeless risks. The HOPE Program is a project initiative designed to assist homeless individuals in New York City with securing and maintaining employment by offering a comprehensive work‐readiness program (The Hope Program, 2023).

In the UK, the updated New Rough Sleeping Strategy was published that £12 m would be allocated to the Test and Learn program to trial innovative approaches and identify what is effective in reducing homelessness and rough sleeping (homeless. org. uk., 2022). The government offers the Future Jobs Fund program to support those at risk or experiencing the homeless (Department for Work and Pensions, 2012). There are various training initiatives aimed at eradicating perpetual homelessness and raising job rates in different countries, but there is an evidence gap in the efficacy of these programs. This review aims to evaluate the impacts of all kinds of skills training for people experiencing or at risk of homelessness and provide policymakers with some guidance.

Previous studies on supporting individuals who are experiencing or at risk of homelessness demonstrated the efficacy of several interventions. Fitzpatrick‐Lewis (2011) evaluated a variety of interventions focused on improving the health and housing status of homeless people, but they did not concentrate on the results linked to employment. Marshall (2021) conducted a systematic review of occupational therapy interventions in the transition from homelessness. The study included two‐arm trials and one‐arm trials, which primarily focused on improving life skills and housing stability. Our review only included the original study that incorporated a control group to avoid selection bias. Employment improvement in the training intervention group is the outcome that we mainly concentrate on. Marshall (2021) included the studies published between 2004 and 2018. Hence, an updated review with high quality is required for us.

## 2 OBJECTIVES

We will solve the following questions:

1. What are the characteristics of skills training services for individuals experiencing or at risk of homelessness?
2. How effectively do the different skills training programs improve employment status, work and life skills, or housing stability?
3. What factors are associated with the variation in the effectiveness of skills training services?

## 3 METHODS

### 3.1 Criteria for considering studies for this review

#### 3.1.1 Types of studies

We will include randomized control trials (RCTs) and quasi‐experimental studies in our review. This inclusive approach acknowledges the limited number of studies employing random assignment in the pre‐search phase for this topic. We will consider both individual random and cluster RCTs that meet the specified inclusion criteria. All included studies should involve the experimental group (participants receiving skills training courses or programs) and the control group (participants with placebo intervention, usual care, or a wait‐list group). The quasi‐experimental studies should contain the pre‐test and post‐test, and report the method used to control the selection bias (such as propensity score matching).

We will exclude studies that lack a control group, such as single‐group before‐after studies. Additionally, studies meeting the inclusion criteria but failing to provide sufficient information for the computation of means and standard deviations for continuous outcomes or event numbers for dichotomous outcomes will be reported for their characteristics but will be excluded from the meta‐analysis due to incomplete data. All studies should be published in English. We will take studies or reports published in peer‐reviewed journals, websites, and gray literature into consideration.

#### 3.1.2 Types of participants

Individuals experiencing and at risk of homelessness will be eligible participants. Homelessness is defined differently across nations and throughout time. We will cite the definition used by Keenan (2021) and O'Leary (2022) in the Campbell Collaboration protocol: Homelessness refers to individuals who do not have access to safe, stable, and adequate housing. This includes those who are living in unsanitary or overcrowded environments, those who are sleeping in public places or on the streets, those who are staying in temporary accommodations such as shelters and hostels, and those who are in insecure living facing eviction or unsafe living conditions. It additionally involves individuals who are residing in inappropriate accommodations, such as tents or vehicles, or who are “sofa surfing” without a fixed address.

Additionally, participants also have to undergo a skills training program that offers comprehensive vocational services to people who are suffering or at risk of homelessness.

#### 3.1.3 Types of interventions

The interventions will provide short‐term or long‐term systematic skills training courses or offer job training opportunities or certifications for individuals experiencing or at risk of homelessness. These courses or training programs aim to improve the following skills of homeless people, including work‐related hard skills (technical skills and work experience), work‐related soft skills (non‐technical skills, such as communication, teamwork, emotional intelligence, etc.), and life skills (fundamental skills, self‐sufficient living skills, and social skills). These courses and training programs could be provided by governments, charitable organizations, faith communities and the non‐profit sector.

#### 3.1.4 Types of outcome measures

We will measure multi‐dimensional outcomes to evaluate the effect of the skills training. We considered employment status as the primary outcome. We regarded work and life skills improvement and housing stability as the secondary outcomes. All included studies must report the primary outcome. We will extract information on secondary outcomes from the included studies, but the absence of secondary outcome reporting will not be a reason for study exclusion.

##### Primary outcomes

Employment status: Any measurement of employment status for every participant after enrolling in the skills training program. Including but not limited to any gainful employment since receiving skills training: such as employment status (full‐ or part‐time work), total income for the previous month, and employment index (Moledina, 2021).

##### Secondary outcomes

- Work and life skills: any measurement of work‐related or life skills following enrollment in the skills training program. Such as communication skills, reading and writing skills, office skills, and social skills.
- Housing stability: any measurement of housing stability, such as the duration of housing stability (Moledina, 2021). The measurement tool is the Residential Timeline Follow‐back Inventory (Tsemberis, 2007).

#### 3.1.5 Duration of follow‐up

We will divide the follow‐up time into short‐term follow‐up (≤1 month), medium‐term follow‐up (1–6 months), and long‐term follow‐up (>6 months).

#### 3.1.6 Types of settings

This systematic review will cover all kinds of training courses and training programs provided by governments, charitable organizations, faith communities, and the non‐profit sectors. Homelessness service settings can be transitional shelters, emergency shelters, respite sites, alternative space programs, or part‐time shelters (Toronto Public Health, 2019). In our systematic review, We will mainly include the following intervention settings:

- Shelter programs: many shelters offer skills training programs to their residents, which can include job skills training, life skills training, and mental health services. These programs can help homeless individuals build the skills they need to become self‐sufficient and find employment.
- Community centers: community centers often offer skills training programs for homeless individuals. These programs can include job training, computer skills training, and other types of vocational training.
- Vocational schools: vocational schools offer specialized training in various trades, such as carpentry, plumbing, and electrician work. These types of programs can provide homeless individuals with the skills they need to find employment in these fields.
- Transitional housing programs: transitional housing programs provide temporary housing and support services to homeless individuals. These programs often include skills training programs, such as job training and life skills training, to help residents become self‐sufficient and transition to permanent housing.
- Nonprofit organizations: nonprofit organizations often offer skills training programs to homeless individuals. These programs can include job training, financial literacy, and other types of skills training to help homeless individuals become self‐sufficient and find employment.
- Online training programs: many online training programs offer skills training courses that homeless individuals can access from anywhere with an internet connection. These programs can include job skills training, computer skills training, and other types of vocational training.

### 3.2 Search methods for identification of studies

The search strategy for PubMed is shown in Supporting Information: Table 1. We first searched keywords in Google Scholar to identify 10 benchmark studies, then compared the final selection results with benchmark studies to evaluate the sensitivity of search strategies. If the sensitivity is not 100%, we will revise the search strategy. We will consult information specialists (Howard White, Yanfei Li, and Information Retrieval Methods Group of Campbell Collaboration) with our information retrieval plan before finalizing the search strategy.

We have conducted a pilot search and will proceed to conduct a systematic search encompassing all retrievable studies and reports, we will also perform manual searches in journals. We plan to check the reference lists of included studies and the related evidence gap map. We will follow the guidance provided by the Campbell Collaboration for search strategy (Kugley, 2017). Studies considered for inclusion will be identified in these three ways:

- The evidence and gap map to improve the welfare of those experiencing and at risk of homelessness (White, 2020).
- Databases: academic databases, evidence and gap map databases, systematic review databases, and trials registry databases.
- Gray literature and websites.

#### 3.2.1 Electronic searches

We will search the following electronic databases with no date, language, or location restrictions:

- Applied Social Sciences Index and Abstracts (ASSIA)
- PubMed.
- Scopus.
- Sociological Abstracts.
- Google Scholar.
- Education Resources Information Center (ERIC)
- Education Abstracts/EducationSource

We will also search the following clinic trial platforms:

- The Cochrane Central Register of Controlled Trials (CENTRAL)
- Clinicaltrials.gov

#### 3.2.2 Searching other resources

We will search the following dissertations, conference proceedings, journals, websites, and gray literature:

- Conference Proceedings Citation Index.
- Networked Digital Library of Theses and Dissertations.
- European Journal of Homelessness.
- Homelessness Studies.
- International Journal of Homelessness.
- Gray Literature Report in 1999 to 2016 (www.greylit.org).
- Homeless Hub https://www.homelesshub.ca/.
- European observatory on homelessness https://www.feantsaresearch.org/en/publications.
- United State interagency council on homelessness http://www.usich.gov/.
- EThOS http://ethos.bl.uk/Home.do.
- WHO ICTRP http://apps.who.int/trialsearch/.
- Focus on Prevention http://www.preventionfocus.net/.
- Social Policy and Practice http://www.spandp.net/.
- 10,000 home campaigns https://en.wikipedia.org/wiki/100,000_Homes_Campaign.
- Anti‐poverty Committee https://en.wikipedia.org/wiki/Anti-Poverty_Committee.
- Back on my feet https://en.wikipedia.org/wiki/Back_on_My_Feet_(non-profit_organization).
- Feantsa https://www.feantsa.org/.
- National Coalition Homeless https://nationalhomeless.org/.
- Homelessness Australia https://www.homelessnessaustralia.org.au/.
- Mission Australia https://www.missionaustralia.com.au/publications/position-statements/homelessness.
- National Alliance to end homelessness https://endhomelessness.org/.
- Institute of global homelessness https://www.ighomelessness.org/.
- Homelessness link https://www.homeless.org.uk/.
- Crisis https://www.crisis.org.uk/about-us/how-we-work/.
- Housing first https://housingfirsteurope.eu/about-the-hub/.
- Canadian Alliance to end homelessness https://housingfirsteurope.eu/about-the-hub/.
- Social work and policy institutes http://www.socialworkpolicy.org/research/homelessness.html.
- Association of housing advice services https://www.ahas.org.uk/.
- Center point https://centrepoint.org.uk/.
- Homelessness trust funds https://housingtrustfundproject.org/htfelements/homeless-trust-funds/.
- Melville charitable trust https://melvilletrust.org/category/resourcesreports/.
- Conrad H Hilton Foundation https://www.hiltonfoundation.org/priorities/homelessness#resources.
- Abt Associates https://www.abtassociates.com/.
- Mathematica https://www.mathematica-mpr.com/.
- American Institutes of Research https://www.air.org/.
- Rand https://www.rand.org/.
- MDRC https://www.mdrc.org/.

### 3.3 Data collection and analysis

#### 3.3.1 Description of methods used in primary research

The primary study should be designed with at least one intervention group and at least one control group. It will use the following study design: RCT or quasi‐experimental design.

#### 3.3.2 Selection of studies

First, we will remove the duplicate publication by Endnote 20 and Rayyan. Second, two reviewers (Z. J. and M. N.) will independently select eligible studies by screening the title and abstract through Endnote 20 and Rayyan software. Third, two authors will rescreen by searching the full text of these studies to determine the final included studies. Two reviewers will resolve the discrepancy by consensus.

#### 3.3.3 Data extraction and management

Two reviews (Z. J. and Y. Q.) will independently extract the following information from each study: the name of the first author, publication year, country, study design (RCT or quasi‐experimental study), inclusion and exclusion criteria, population type (experiencing homelessness or at risk of homelessness), the characteristics of enrolled subjects (number, age, gender), type of intervention group (work‐related hard skills training, work‐related soft skills training or life skills training), intervention goals, type of control group (placebo intervention, usual care or at wait‐list), intervention settings, intervention duration, intervention intensity, intervention adaptation, intervention integrity, and the effect size for intervention. We also need to extract the baseline and final values or any changes in the focus outcomes.

#### 3.3.4 Assessment of risk of bias in included studies

For the studies that met the inclusion of this review, RCTs will be assessed by the Cochrane Risk of Bias tool 2.0 (ROB 2.0) (Sterne, 2019). Quasi‐experimental studies will be evaluated by the Cochrane Risk of Bias In Non‐randomized Studies–of Interventions tool (ROBINS‐I) (Sterne, 2016). Two reviewers (M. Y. and K. G.) will independently evaluate the quality. The discrepancies will be resolved by consensus. The ROB 2.0 tools have the following aspects: (1) bias arising from the randomization process; (2) bias due to deviations from intended interventions; (3) bias due to missing outcome data; (4) bias in the measurement of the outcome; (5) bias in the selection of the reported result. The studies will reach an overall judgment about the risk of bias as “low risk of bias,” “some concerns” and “high risk of bias.” The ROBINS‐I tools have 7 domains including (1) bias due to confounding; (2) bias in the selection of participants into the study; (3) bias in the classification of interventions; (4) bias due to deviations from intended interventions; (5) bias due to missing data; (6) bias in the measurement of outcomes; (7) bias in the selection of the reported result. We can judge the overall risk of bias for every study as “low risk of bias,” “moderate risk of bias,” “serious risk of bias,” “critical risk of bias” and “no information.”

We will present the assessment results of RCTs and quasi‐experimental studies separately. Additionally, we plan to perform sensitivity analyses distinguishing the role of low and high risk of bias, we will conduct separate sensitivity analyses for different study designs and distinct measures of treatment effect.

We will extract the standardized effect sizes from the study to perform the meta‐analysis. For the studies without standardized effect sizes, we plan to use the Campbell Collaboration's effect size calculator. If there is insufficient information to calculate the effect size, we will contact the author to access the related data. If it is impossible to acquire detailed information, we will describe the characteristics but exclude the study in the synthesis of the results. For dichotomous outcomes, such as employment status, and housing status, we will use odds ratios (ORs) with 95% confidence intervals (CIs). For continuous outcomes, such as skills score, we will use the mean differences (MDs) with 95% CI if they are reported in the same measurement scale. With the continuous outcomes reported in the different scales, we plan to calculate Hedges' *g* to report standardized mean differences (SMDs) in order to avoid overestimation (Hedges, 1981).

#### 3.3.5 Unit of analysis issues

We recognize that one or more interventions may be evaluated in a single study. In this case, the pair‐wise comparison should be assessed separately. For trials with three‐arms or multi‐arms, we plan to use the three‐level model or the Robust Variance Estimation method to incorporate several effect sizes within studies (Noortgate, 2014; Tipton, 2015). If there is more than one intervention duration, we will regard each duration as an independent group. For the cluster‐randomized trials, we will assess the unit of analysis errors. If there is a unit of errors, we need to use an intra‐cluster correlation coefficient collected from related trials to inflate the standard deviation (Li, 2022).

#### 3.3.6 Criteria for determination of independent findings

There is a risk of overlapping results between studies. Therefore, to avoid counting data repeatedly, we will compare the studies that met the inclusion criteria for the authors, affiliation institution, inclusion criteria, and data details. We will only include the unique data from each study.

#### 3.3.7 Dealing with missing data

We will try to contact the author to obtain all the data when we encounter a problem with missing data. If it is unable to access the necessary data of the included study, especially effect size data, we will report the characteristics of the study but state that it could not be included in the meta‐analysis because of missing data.

We plan to use scatterplots and boxplots to identify outliers. If we detect an outlier, we need to analyze whether it is caused by a measurement error or a data entry error, then correct the error if possible. We plan to carry out a sensitivity analysis and report both the results with the outlier and without the outlier.

#### 3.3.8 Assessment of heterogeneity

There are two ways to assess the heterogeneity of the included studies. We will use the *I* ^2^ statistic (range: 0%–100%) and the likelihood ratio test to assess heterogeneity. The *I* ^2^ in the range of 0% to 40% is regarded as not important; 30% to 60% represents moderate heterogeneity; 50% to 90% represents substantial heterogeneity; 75% to 100% is regarded as considerable heterogeneity (Higgins, 2023). The likelihood‐based method has a consideration of between‐studies variance. This method is preferred to the standard method in undertaking random effects meta‐analysis when the value of between‐trial components of variance has an important effect on the overall estimated treatment effect (Hardy, 1996).

#### 3.3.9 Assessment of reporting biases

Funnel plots and Egger's test can be performed to search for the signs of asymmetry to examine the publication bias if the number of included studies is 10 or more. We will not evaluate publication bias when there are fewer than 10 studies with the same endpoint. Less than 10 studies will not properly distinguish chance from real asymmetry.

#### 3.3.10 Data synthesis

All analyses will be conducted using the statistical programming language R, package *metafor*. We will pool data from the same endpoint. For dichotomous outcomes, we will use the DerSimonian‐Laird inverse variance method and the random effect model to combine studies by ORs with corresponding 95% CIs. For continuous outcomes, we will combine studies by MD or SMD with a corresponding 95% CIs. If it couldn't be performed in a quantitative synthesis, study findings will be synthesized narratively (Campbell, 2020).

#### 3.3.11 Subgroup analysis and investigation of heterogeneity

There are considerable heterogeneities between the interventions, the measurement of outcomes, and the measurement time points related to skills training. We will perform subgroup analysis by meta‐regression in the following scenarios.

- Demographics (age, sex, ethnicity)
- Study design
- Critical appraisal
- Training program
- Intervention location
- Time effect
- Intervention duration
- Intervention integrity

#### 3.3.12 Sensitivity analysis

We will perform sensitivity analyses to assess the robustness of our findings. These analyses will include the isolation of studies based on their study design, distinguishing RCTs and quasi‐experimental designs in the pooling process. We will also conduct sensitivity analyses to differentiate the influence of low and high risk of bias. In cases where we identify an outlier, we will make separate data pools, one including the outlier and one excluding it (O'Leary, 2022).

#### 3.3.13 Treatment of qualitative research

We do not plan to include qualitative research.

#### 3.3.14 Summary of findings and assessment of the certainty of the evidence

We do not plan to include a summary of findings and an assessment of the certainty of the evidence.

## CONTRIBUTIONS OF AUTHORS

- Content: Zijun Li, Mina Ma, Kehu Yang
- Systematic review methods: Zijun Li, Yongqi Yang
- Statistical analysis: Minyan Yang, Ke Guo
- Information retrieval: Zijun Li, Yanfei Li, Howard White

## DECLARATIONS OF INTEREST

There is no conflicts of interests.

## SOURCES OF SUPPORT

### Internal sources

- the Major Project of the National Social Science Fund of China, China

“Research on the Theoretical System, International Experience and Chinese Path of Evidence‐based Social Science” (Project No. 19ZDA142).

### External sources

- No sources of support provided

## Supporting information

Supporting information.

## References

[cl21372-bib-0002] Aldridge, R. W. , Story, A. , Hwang, S. W. , Nordentoft, M. , Luchenski, S. A. , Hartwell, G. , Tweed, E. J. , Lewer, D. , Vittal Katikireddi, S. , & Hayward, A. C. (2018). Morbidity and mortality in homeless individuals, prisoners, sex workers, and individuals with substance use disorders in high‐income countries: A systematic review and meta‐analysis. The Lancet, 391(10117), 241–250. 10.1016/S0140-6736(17)31869-X PMC5803132 29137869

[cl21372-bib-0004] Beijer, U. , Wolf, A. , & Fazel, S. (2012). Prevalence of tuberculosis, hepatitis C virus, and HIV in homeless people: A systematic review and meta‐analysis. The Lancet Infectious Diseases, 12(11), 859–870. 22914343 10.1016/S1473-3099(12)70177-9 PMC3494003

[cl21372-bib-0005] Bernstein, R. S. , Meurer, L. N. , Plumb, E. J. , & Jackson, J. L. (2015). Diabetes and hypertension prevalence in homeless adults in the United States: A systematic review and meta‐analysis. American Journal of Public Health, 105(2), e46–e60. 10.2105/AJPH.2014.302330 PMC4318300 25521899

[cl21372-bib-0007] Campbell, M. , McKenzie, J. E. , Sowden, A. , Katikireddi, S. V. , Brennan, S. E. , Ellis, S. , Hartmann‐Boyce, J. , Ryan, R. , Shepperd, S. , Thomas, J. , Welch, V. , & Thomson, H. (2020). Synthesis without meta‐analysis (SWiM) in systematic reviews: Reporting guideline. BMJ, 368, l6890. 31948937 10.1136/bmj.l6890 PMC7190266

[cl21372-bib-0009] Department for Work and Pensions . (2012). Impacts and costs and benefits of the future jobs fund. https://assets.publishing.service.gov.uk/media/5a7c00bde5274a7318b906f1/impacts_costs_benefits_fjf.pdf

[cl21372-bib-0011] eSoft Skill . (2023). Soft skills vs life skills. https://esoftskills.com/soft-skills-vs-life-skills/

[cl21372-bib-0012] Fitzpatrick‐Lewis, D. , Ganann, R. , Krishnaratne, S. , Ciliska, D. , Kouyoumdjian, F. , & Hwang, S. W. (2011). Effectiveness of interventions to improve the health and housing status of homeless people: A rapid systematic review. BMC Public Health, 11(1), 638. 21831318 10.1186/1471-2458-11-638 PMC3171371

[cl21372-bib-0013] Frankish, C. J. , Hwang, S. W. , & Quantz, D. (2005). Homelessness and health in Canada: Research lessons and priorities. Canadian Journal of Public Health, 96(Suppl 2), S23–S29. 16078553 10.1007/BF03403700 PMC6976130

[cl21372-bib-0016] GCFGlobal . (2023). Job search and networking – Hard Skills vs. Soft Skills. https://edu.gcfglobal.org/en/jobsearchandnetworking/hard-skills-vs-soft-skills/1/

[cl21372-bib-0017] Glass, R. , Sevitz, B. , Williamson, S. , Wink, S. , & Duncan, M. (2006). The occupational needs of homeless people at a place of renewal in Cape Metropole. South African Journal of Occupational Therapy, 36(1), 11–13.

[cl21372-bib-0018] Guha, M. (2008). Homelessness handbook. Reference Reviews, 22(1), 22.

[cl21372-bib-0019] Gutwinski, S. , Schreiter, S. , Deutscher, K. , & Fazel, S. (2021). The prevalence of mental disorders among homeless people in high‐income countries: An updated systematic review and meta‐regression analysis. PLoS Medicine, 18(8), e1003750. 34424908 10.1371/journal.pmed.1003750 PMC8423293

[cl21372-bib-0020] Hardy, R. J. , & Thompson, S. G. (1996). A likelihood approach to meta‐analysis with random effects. Statistics in Medicine, 15(6), 619–629. 8731004 10.1002/(SICI)1097-0258(19960330)15:6<619::AID-SIM188>3.0.CO;2-A

[cl21372-bib-0021] Hedges, L. V. (1981). Distribution theory for Glass's estimator of effect size and related estimators. Journal of Educational and Behavioral Statistics, 6, 107–128.

[cl21372-bib-0022] Higgins, J. P. T. , Thomas, J. , Chandler, J. , Cumpston, M. , Li, T. , Page, M. J. , & Welch, V. A. (Eds.). (2023). Cochrane Handbook for Systematic Reviews of Interventions version 6.4 (updated August 2023). Cochrane.

[cl21372-bib-0023] Homeless Hub . (2023). Life skills training. https://www.homelesshub.ca/about-homelessness/service-provision/life-skills-training

[cl21372-bib-0024] Homeless Hub . (2021). Employment training. https://www.homelesshub.ca/solutions/education-training-and-employment/training

[cl21372-bib-0025] homeless.org.uk . (2022). A summary of the Government's new rough sleeping strategy. https://homeless.org.uk/news/a-summary-of-the-governments-new-rough-sleeping-strategy/

[cl21372-bib-0026] Keenan, C. , Sarah, M. , Jennifer, H. , Terri, P. , Jayne, H. , Christopher, C. , Peter, M. , Suzanne, F. , & John, C. (2021). Accommodation‐based interventions for individuals experiencing, or at risk of experiencing, homelessness. Campbell Systematic Reviews, 17(2), e1165. 37131929 10.1002/cl2.1165 PMC8356295

[cl21372-bib-0028] Kugley, S. , Wade, A. , Thomas, J. , Mahood, Q. , Klint Jorgensen, A. M. , Hammerstrom, K. , & Sathe, N. (2017). Searching for studies: A guide to information retrieval for Campbell systematic reviews. The Campbell Collaboration.

[cl21372-bib-0029] Labour and Social Affairs . (2021). Homeless population. https://www.oecd.org/els/family/HC3-1-Homeless-population.pdf

[cl21372-bib-0031] Li, Y. , Li, X. , Li, R. , Chen, N. , & Yang, K. (2022). PROTOCOL: Home‐based care for people with dementia: A systematic review. Campbell Systematic Reviews, 18(4), e1285. 36908844 10.1002/cl2.1285 PMC9629276

[cl21372-bib-0032] Marshall, C. A. , Leonie, B. , Ann, W. L. , Roxanne, I. , & Gutman Sharon, A. (2021). A systematic review of occupational therapy interventions in the transition from homelessness. Scandinavian Journal of Occupational Therapy, 28(3), 171–187. 32476575 10.1080/11038128.2020.1764094

[cl21372-bib-0035] Moledina, A. , Olivia, M. , Eric, A. , Jui‐Hsia, H. , Ammar, S. , Kednapa, T. , Ginetta, S. , Gary, B. , David, P. , Tim, A. , Claire, K. , & Pottie, K. (2021). A comprehensive review of prioritised interventions to improve the health and wellbeing of persons with lived experience of homelessness. Campbell Systematic Reviews, 17(2), e1154. 37131928 10.1002/cl2.1154 PMC8356292

[cl21372-bib-0036] National Alliance to End Homelessness . (2016). The state of homelessness in America. https://endhomelessness.org/

[cl21372-bib-0038] Noortgate, W. V. d. , López‐López, J. A. , Marín‐Martínez, F. , & Sánchez‐Meca, J. (2014). Meta‐analysis of multiple outcomes: A multilevel approach. Behavior Research Methods, 47, 1274–1294. 10.3758/s13428-014-0527-2 25361866

[cl21372-bib-0039] O'Leary, C. , Anton, R. , Ligia, T. , & Esther, C. (2022). PROTOCOL: The experiences of adults experiencing homelessness when accessing and using psychosocial interventions: A systematic review and qualitative evidence synthesis. Campbell Systematic Reviews, 18(4), e1289. 36908840 10.1002/cl2.1289 PMC9683077

[cl21372-bib-0043] Stephen, G. , & O'Grady, B (2013). Youth homelessness in Canada: Implications for policy and practice. Why don't you just get a job? Homeless Youth, Social Exclusion and Employment Training.

[cl21372-bib-0044] Sterne, J. A. , Hernán, M. A. , Reeves, B. C. , Savović, J. , Berkman, N. D. , Viswanathan, M. , Henry, D. , Altman, D. G. , Ansari, M. T. , Boutron, I. , Carpenter, J. R. , Chan, A. W. , Churchill, R. , Deeks, J. J. , Hróbjartsson, A. , Kirkham, J. , Jüni, P. , Loke, Y. K. , Pigott, T. D. , … Higgins, J. P. (2016). ROBINS‐I: A tool for assessing risk of bias in non‐randomised studies of interventions. BMJ, 355, i4919. 27733354 10.1136/bmj.i4919 PMC5062054

[cl21372-bib-0045] Sterne, J. A. C. , Savović, J. , Page, M. J. , Elbers, R. G. , Blencowe, N. S. , Boutron, I. , Cates, C. J. , Cheng, H. Y. , Corbett, M. S. , Eldridge, S. M. , Emberson, J. R. , Hernán, M. A. , Hopewell, S. , Hróbjartsson, A. , Junqueira, D. R. , Jüni, P. , Kirkham, J. J. , Lasserson, T. , Li, T. , … Higgins, J. P. T. (2019). RoB 2: A revised tool for assessing risk of bias in randomised trials. BMJ, 366, l4898. 31462531 10.1136/bmj.l4898

[cl21372-bib-0047] The Hope Program . (2023). How it works. https://www.thehopeprogram.org/

[cl21372-bib-0048] Tipton, E. (2015). Small sample adjustments for robust variance estimationwith meta‐regression. Psychological Methods, 20, 375–393. 24773356 10.1037/met0000011

[cl21372-bib-0049] Toronto Public Health . (2019). Infection prevention and control guide for homelessness service settings. https://www.toronto.ca/wp-content/uploads/2019/09/98bf-tph-infection-prevention-and-control-homeless-service-settings-2019-.pdf

[cl21372-bib-0050] Tsemberis, S. , Gregory, M. H. , Valerie, W. , Hanrahan, P. , & Stefancic, A. (2007). Measuring homelessness and residential stability: The residential time‐line follow‐back inventory. Journal of Community Psychology, 35(1), 29–42.

[cl21372-bib-0051] United Nations . (2023). Universal Declaration of Human Rights. https://www.un.org/en/about-us/universal-declaration-of-human-rights

[cl21372-bib-0052] US Department of Labor . (2023). Workforce Innovation and Opportunity Act. https://www.dol.gov/agencies/eta/wioa

[cl21372-bib-0053] Veterans' Employment and Training Service . (2023). About VETS. https://www.dol.gov/agencies/vets

[cl21372-bib-0054] Wallis, J. J. (1988). A caring society: The New Deal, the Worker, and the Great Depression. By Irving Bernstein. Boston: Houghton‐Mifflin Company, 1985. Pp. xii, 338. $22.95. The Journal of Economic History, 48(1), 221–223.

[cl21372-bib-0055] White, H. , Saran, A. , Verma, A. , & Verma, K. (2020). The effectiveness of interventions to improve the welfare of those experiencing and at risk of homelessness: An updated evidence and gap map. Global Evidence and Gap Map of Effectiveness, Third Edition. Centre for Homelessness Impact, Campbell Collaboration & Heriot Watt University.

[cl21372-bib-0056] Williams, S. P. , & Bryant, K. L. (2018). Sexually transmitted infection prevalence among homeless adults in the United States: A systematic literature review. Sexually Transmitted Diseases, 45(7), 494–504. 29465661 10.1097/OLQ.0000000000000780 PMC6367672

[cl21372-bib-0058] Other published versions of this review

[cl21372-bib-0059] Classification pending references

