# Supplementary material for: PROTOCOL: The effectiveness of skills training to increase employment among those experiencing and at risk of homelessness: A systematic review
Source: Campbell Syst Rev. 2023 Dec 9;19(4):e1372. doi: 10.1002/cl2.1372 (PMC10710540; doi:10.1002/cl2.1372)
Supplement: Supplementary file 1 — Supporting information. [file CL2-19-e1372-s001.docx]

# Appendices

## 1 Supplementary Table 1: Search Strategy for PubMed

EmploymentWe will search PubMed, The Cochrane Central Register of Controlled Trials (CENTRAL), the Applied Social Sciences Index and Abstracts (ASSIA), Scopus, Sociological Abstracts, Google Scholar, Education Resources Information Center (ERIC), and Education Abstracts/EducationSource. We will also search the clinic registry platform: The Cochrane Central Register of Controlled Trials (CENTRAL) and Clinicaltrials.gov. Supplementary Table 1 shows the search strategy for PubMed.

| Search Number | Search Strategy for PubMed |
| --- | --- |
| 1 | "Homeless Youth"[MeSH Terms] |
| 2 | "Ill-Housed Persons"[MeSH Terms] |
| 3 | "evicted"[Title/Abstract] OR "eviction"[Title/Abstract] OR "homeless*"[Title/Abstract] OR "housing exclusion"[Title/Abstract] OR "living on the street*"[Title/Abstract] OR "residential stability"[Title/Abstract] OR "stable housing"[Title/Abstract] OR "street dwelling"[Title/Abstract] OR "street dwellers"[Title/Abstract] OR "non private dwelling"[Title/Abstract] OR "shelter dwellers"[Title/Abstract] OR "street life"[Title/Abstract] OR "street youth"[Title/Abstract] OR "street children"[Title/Abstract] OR "street people"[Title/Abstract] OR "marginally housed"[Title/Abstract] OR "precarious housing"[Title/Abstract] OR "housing first"[Title/Abstract] OR "runaway*"[Title/Abstract] OR "bag lady"[Title/Abstract] OR "houseless*"[Title/Abstract] OR "Unhoused"[Title/Abstract] OR "Roofless"[Title/Abstract] OR "rough sleep*"[Title/Abstract] OR "destitute*"[Title/Abstract] OR "skid row*"[Title/Abstract] OR "street people"[Title/Abstract] OR "street person*"[Title/Abstract] OR "street youth"[Title/Abstract] OR "street child*"[Title/Abstract] OR "street life"[Title/Abstract] OR "street living"[Title/Abstract] OR "sleeping rough"[Title/Abstract] OR "emergency accommodation"[Title/Abstract] OR "temporary accommodation"[Title/Abstract] OR "insecure accommodation"[Title/Abstract] OR Unsheltered[Title/Abstract] OR "couch surfing"[Title/Abstract] OR  "sofa surfing"[Title/Abstract] OR "without home*"[Title/Abstract] OR "without hous*"[Title/Abstract] OR ill-housed[Title/Abstract] OR illhoused[Title/Abstract]. |
| 4 | Skills training [MeSH] OR Vocational education [MeSH] OR Employment [MeSH] OR Occupations [MeSH] OR Work [MeSH] OR Skills [Title/Abstract] OR employment [Title/Abstract] OR job [Title/Abstract] OR jobs [Title/Abstract] OR trade [Title/Abstract] OR trades [Title/Abstract] OR vocation* [Title/Abstract] OR occupation*[Title/Abstract] OR career* [Title/Abstract] OR work [Title/Abstract] |
| 5 | Education [MeSH] OR Training support [MeSH] OR education [Title/Abstract] OR training [Title/Abstract] OR program* [Title/Abstract] OR Intervention* [Title/Abstract] |
| 6 | #1 OR #2 OR #3 |
| 7 | #4 AND #5 |
| 8 | #6 AND #7 |
| 9 | "Employment"[MeSH Terms] OR "occupations"[MeSH Terms] OR "work"[MeSH Terms] OR "occupations"[MeSH Terms] |
| 10 | "employ*"[Title/Abstract] OR "pre employ*"[Title/Abstract] OR "work*"[Title/Abstract] OR "vocation*"[Title/Abstract] OR "occupation*"[Title/Abstract] OR "trade*"[Title/Abstract] OR "career*"[Title/Abstract] OR "job"[Title/Abstract] OR "skill*"[Title/Abstract] |
| 11 | #9 OR #10 |
| 12 | #8 AND #11 |

## 2 Supplementary 2: Screening tool and data extraction of three rounds of pilots

Table 1 Screening Tool

| Study ID | Language | Study design | Population | Intervention | Outcome | Decision |
| --- | --- | --- | --- | --- | --- | --- |
| (Poremski & Hwang, 2016) | Yes | No | Yes | Yes | Yes | Exclude |
| (Taib et al., 2022) | Yes | No | Yes | Yes | Yes | Exclude |
| (Zhang & Slesnick, 2018) | Yes | Yes | Yes | No | Yes | Exclude |
| (Lenzi et al., 2021) | Yes | Yes | No | No | Yes | Exclude |
| (Meade & Slesnick, 2002) | Yes | No | Yes | No | Yes | Exclude |
| (Shulman et al., 2018) | Yes | Yes | No | Yes | Yes | Exclude |
| (Kidd et al., 2020) | Yes | Yes | Yes | No | Yes | Exclude |
| (Gabrielian et al., 2019) | Yes | Yes | Yes | No | No | Exclude |
| (Brown et al., 2019) | Yes | No | Yes | No | Yes | Exclude |
| (Wang, 2019) | Yes | No | No | No | Yes | Exclude |
| (LePage, 2021) | Yes | Yes | Yes | Yes | Yes | Include |
| (Nelson et al., 2012) | Yes | No | Yes | Yes | Yes | Exclude |
| (Barber et al., 2005) | Yes | No | Yes | Yes | Yes | Exclude |
| (Elbogen., 2021) | Yes | No | Yes | No | Yes | Exclude |
| (Kaltsidis, 2021) | Yes | Yes | Yes | No | No | Exclude |
| (Gao et al., 2016) | Yes | Yes | Yes | Yes | No | Exclude |
| (Marshall, 2022) | Yes | No | Yes | Yes | Yes | Exclude |

Table 2 Excluded Studies

| Study | Reason for exclusion |
| --- | --- |
| (Poremski & Hwang, 2016) | Wrong study design |
| (Taib et al., 2022) | Wrong study design |
| (Zhang & Slesnick, 2018) | Wrong intervention |
| (Lenzi et al., 2021) | Wrong population and wrong intervention |
| (Meade & Slesnick, 2002) | Wrong study design and wrong intervention |
| (Shulman et al., 2018) | Wrong population |
| (Gabrielian et al., 2019) | Wrong outcome and wrong intervention |
| (Kidd et al., 2020) | Wrong intervention |
| (Brown et al., 2019) | Wrong study design and wrong intervention |
| (Wang, 2019) | Wrong population and wrong intervention |
| (Elbogen., 2021) | Wrong study design and wrong intervention |
| (Kaltsidis, 2021) | Wrong intervention and wrong outcome |
| (Gao et al., 2016) | Wrong outcome |
| (Marshall, 2022) | Wrong study design |
| (Nelson, 2012) | Wrong study design |
| (Barber, 2005) | Wrong study design |

Table 3 Standardized data collection

| Title |
| --- |
| First Author |
| Year of Publication |
| Journal |
| Language |
| Sample Characteristics: Age, Ethics, Region, Numb |
| Intervention Information: Study design, Training program, Intervention duration, Comparison, Follow-up |
| Outcomes: Outcome, Measurement, Subgroup, Subgroup Variable, Effect index, Sample size, Effect size. |

Table 4 Data extraction of three rounds of pilots

| **(LePage, 2021)** | |
| --- | --- |
| **Basic Information** | -Title: Individualized Placement and Support Supported Employment for Justice-involved Homeless and Unemployed Veterans  -First Author: LePage, James  -Year of Publication: 2021  -Journal: LePage, James  -Language: English |
| **Sample Characteristics** | -Age: AFVP: 51.1 (5.0)；AFVP+IPS-SE: 51.7 (6.5)  -ethnic minority: AFVP: 72%; AFVP+IPS-SE: 78.9%  -Region: America |
| **Intervention Information** | - Study design: prospective cohort study  - training program: Individualized Placement and Support, Supported Employment program+ About Face Vocational Program  - Intervention Duration: 27.32 (SD=28.22) days  - Comparison: People attend the About Face Vocational Program  - Follow-up: 6 months |
| **Outcomes** | Outcome: Employed at 180 days  Sample Size = 63  Intervention: n=38  (Pre) n=0, P=0%  (Post) n=21, P=55.3%  Comparison: n=25  (Pre) n=0, P=0%  (Post) n=6, P=24%  Outcome: Housed at 180 d  Intervention: n=38  (Pre) n=0, P=0%  (Post) n=22, P=57.9%  Comparison: n=25  (Pre) n=0, P=0%  (Post) n=15, P=52% |

Excluded studies Reference.

Brown, M., Mihelicova, M., Collins, K., & Ponce, A. (2019). Predictors of employment outcomes in a comprehensive service program for individuals experiencing chronic homelessness. *Am J Orthopsychiatry*, *89*(2), 279-286. <https://doi.org/10.1037/ort0000358>

Elbogen, E. B., Lanier, M., Wagner, H. R., & Tsai, J. (2021). Financial Strain, Mental Illness, and Homelessness: Results From a National Longitudinal Study. Med Care, 59(Suppl 2), S132-s138. https://doi.org/10.1097/mlr.0000000000001453

Gabrielian, S., Bromley, E., Hamilton, A. B., Vu, V. T., Alexandrino, A., Koosis, E., & Young, A. S. (2019). Problem-solving skills and deficits among homeless veterans with serious mental illness. Am J Orthopsychiatry, 89(2), 287-295. <https://doi.org/10.1037/ort0000340>

Gao, N., Dolce, J., Rio, J., Heitzmann, C., & Loving, S. (2016). In-vivo job development training among peer providers of homeless veterans supported employment programs. *Psychiatr Rehabil J*, *39*(2), 191-192. <https://doi.org/10.1037/prj0000196>

Kaltsidis, G., Grenier, G., Cao, Z., L'Espérance, N., & Fleury, M. J. (2021). Typology of changes in quality of life over 12 months among currently or formerly homeless individuals using different housing services in Quebec, Canada. Health Qual Life Outcomes, 19(1), 128. https://doi.org/10.1186/s12955-021-01768-y

Kidd, S. A., Vitopoulos, N., Frederick, T., Leon, S., Wang, W., Mushquash, C., & McKenzie, K. (2020). Trialing the feasibility of a critical time intervention for youth transitioning out of homelessness. American Journal of Orthopsychiatry, 90, 535-545. https://doi.org/10.1037/ort0000454

Lenzi, M., Santinello, M., Gaboardi, M., Disperati, F., Vieno, A., Calcagnì, A., Greenwood, R. M., Rogowska, A. M., Wolf, J. R., Loubière, S., Beijer, U., Bernad, R., Vargas-Moniz, M. J., Ornelas, J., Spinnewijn, F., & Shinn, M. (2021). Factors Associated with Providers' Work Engagement and Burnout in Homeless Services: A Cross-national Study. Am J Community Psychol, 67(1-2), 220-236. https://doi.org/10.1002/ajcp.12470

Meade, M. A., & Slesnick, N. (2002). Ethical considerations for research and treatment with runaway and homeless adolescents. J Psychol, 136(4), 449-463. https://doi.org/10.1080/00223980209604171

Poremski, D., & Hwang, S. W. (2016). The Willingness of Housing First Participants to Consider Supported-Employment Services. Psychiatr Serv, 67(6), 667-670. https://doi.org/10.1176/appi.ps.201500140

Shulman, C., Hudson, B. F., Kennedy, P., Brophy, N., & Stone, P. (2018). Evaluation of training on palliative care for staff working within a homeless hostel. Nurse Educ Today, 71, 135-144. https://doi.org/10.1016/j.nedt.2018.09.022

Taib, N. I., Öster, C., & Ramklint, M. (2022). Former street-working boys in Iraq highlight the importance of receiving education, training, and support from families and other adults. Acta Paediatr, 111(7), 1390-1398. https://doi.org/10.1111/apa.16315

Wang, J. Z., Mott, S., Magwood, O., Mathew, C., McLellan, A., Kpade, V., Gaba, P., Kozloff, N., Pottie, K., & Andermann, A. (2019). The impact of interventions for youth experiencing homelessness on housing, mental health, substance use, and family cohesion: a systematic review. BMC Public Health, 19(1), 1528. https://doi.org/10.1186/s12889-019-7856-0

Zhang, J., & Slesnick, N. (2018). Substance use and social stability of homeless youth: A comparison of three interventions. Psychol Addict Behav, 32(8), 873-884. https://doi.org/10.1037/adb0000424

Marshall, C. A., Boland, L., Westover, L. A., Goldszmidt, R., Bengall, J., Aryobi, S., Isard, R., Easton, C., & Gewurtz, R. (2022). Effectiveness of employment-based interventions for persons experiencing homelessness: A systematic review. *Health Soc Care Community*, *30*(6), 2142-2169. <https://doi.org/10.1111/hsc.13892>

Barber, C. C., Fonagy, P., Fultz, J., Simulinas, M., & Yates, M. (2005). Homeless near a thousand homes: outcomes of homeless youth in a crisis shelter. *Am J Orthopsychiatry*, *75*(3), 347-355. <https://doi.org/10.1037/0002-9432.75.3.347>

Nelson, S. E., Gray, H. M., Maurice, I. R., & Shaffer, H. J. (2012). Moving ahead: evaluation of a work-skills training program for homeless adults. Community Ment Health J, 48(6), 711-722. <https://doi.org/10.1007/s10597-012-9490-5>

Included studies Reference.

LePage, J. P., Martin, W. B., Crawford, A. M., Rock, A., Parish Johnson, J. A., & Washington, E. L. (2021). Individualized Placement and Support Supported Employment for Justice-involved Homeless and Unemployed Veterans. Med Care, 59(Suppl 2), S195-s198. <https://doi.org/10.1097/mlr.0000000000001445>
